# Associations between maternal and offspring glucose metabolism: a 9-year follow-up of a randomised controlled trial

**DOI:** 10.3389/fendo.2023.1324925

**Published:** 2024-01-10

**Authors:** Sigrid L. Nyen, Astrid Kamilla Stunes, Kari Anne I. Evensen, Torunn Børsting, Unni Syversen, Kjell Å. Salvesen, Siv Mørkved, Signe N. Stafne

**Affiliations:** ^1^ Faculty of Medicine and Health Sciences, Norwegian University of Science and Technology (NTNU), Trondheim, Norway; ^2^ Department of Clinical and Molecular Medicine, Faculty of Medicine and Health Sciences, Norwegian University of Science and Technology (NTNU), Trondheim, Norway; ^3^ Center for Oral Health Services and Research, Mid-Norway (TkMidt), Trondheim, Norway; ^4^ Children’s Clinic, St. Olavs Hospital, Trondheim University Hospital, Trondheim, Norway; ^5^ Department of Rehabilitation Science and Health Technology, Faculty of Health Sciences, Oslo Metropolitan University, Oslo, Norway; ^6^ Department of Public Health and Nursing, Faculty of Medicine and Health Sciences, Norwegian University of Science and Technology (NTNU), Trondheim, Norway; ^7^ Department of Endocrinology, St. Olavs Hospital, Trondheim University Hospital, Trondheim, Norway; ^8^ Department of Obstetrics and Gynecology, St. Olavs Hospital, Trondheim University Hospital, Trondheim, Norway; ^9^ Clinic of Rehabilitation, St. Olavs Hospital, Trondheim University Hospital, Trondheim, Norway

**Keywords:** blood glucose, child, follow-up, gestational diabetes, hyperglycaemia, insulin resistance, prenatal exposure

## Abstract

**Introduction:**

There is increasing evidence that the *in utero* environment affects the health and disease risk of offspring throughout their lives. The long-term effect of maternal hyperglycaemia on offspring glucose metabolism is of interest in a public health perspective. The aim of this study was to examine the association between *in utero* exposure to maternal glycaemia and offspring glucose metabolism.

**Methods:**

Mother-child pairs were recruited from an RCT to prevent gestational diabetes mellitus where 855 healthy pregnant women were randomised to exercise or standard antenatal care. The original RCT detected no group differences in gestational diabetes mellitus prevalence or insulin resistance. The two groups were analysed as one group in the present study. Maternal glucose levels were assessed after 2-hour 75-gram oral glucose tolerance tests in pregnancy week ~34. Offspring outcomes were evaluated at ~9 years of age and included fasting glucose and homeostatic model assessment of insulin resistance. Multivariable regression models were performed, controlling for potential hereditary and lifestyle confounding factors.

**Results:**

Complete data were available for 105 mother-child pairs. The regression analysis showed a positive association between maternal and offspring fasting glucose that was borderline significant (beta=0.18, 95% CI [-0.00027, 0.37], p=0.050). We did not find significant associations between maternal fasting glucose and offspring insulin resistance (beta=0.080, 95% CI [-0.087, 0.25], p=0.34), or between maternal 2-hour glucose and offspring fasting glucose (beta=0.016, 95% CI [-0.038, 0.070], p=0.56) or insulin resistance (beta=0.017, 95% CI [-0.032, 0.065], p=0.49).

**Conclusions:**

Assessing a homogeneous group of healthy mother-child pairs, we found a borderline significant positive association between maternal and offspring fasting glucose, which persisted after adjustment for potential hereditary and lifestyle confounding factors. Our findings support other similar studies and highlight that improving the metabolic health of pregnant women, and women in childbearing age, should remain a key public health priority.

**Clinical trial registration:**

ClinicalTrials.gov, identifier NCT00476567.

## Introduction

There is increasing evidence that the *in utero* environment affects the health and disease risk of offspring throughout their lives (1–3). Hyperglycaemia and gestational diabetes mellitus (GDM) are common pregnancy complications, and consequences of different glycaemic levels in pregnancy have been widely studied. It is well established that GDM increases the risk of future type 2 diabetes in the mother and adverse pregnancy outcomes (4–6). Studies also indicate potential long-term effects on the offspring, including obesity, hypertension, and abnormal glucose metabolism (7–13). These associations are, however, less clear, and susceptible to confounders due to the long-term perspective and multifactorial aetiology.

The incidence and prevalence of type 2 diabetes among children and adolescents are increasing (14, 15), and associations between maternal glucose metabolism and offspring risk of childhood diabetes have been investigated. A large Canadian retrospective cohort study reported higher incidence of diabetes in offspring of GDM mothers, when adjusted for lifestyle confounders, but not hereditary factors (16). However, a systematic review by Kawasaki et al. did not detect increased risk of diabetes in children of mothers with GDM (10).

Childhood glucose metabolism is a more frequently studied outcome than childhood diabetes. As this is a strong predictor of future risk of type 2 diabetes (17–19), it is a highly relevant outcome to assess. The relationship between maternal and offspring glucose metabolism has been examined in multiple studies, but the conclusions are divergent, possibly due to methodologic differences and quality. Two systematic reviews reported an association between GDM and offspring glucose metabolism, but systematic adjustments for potential hereditary and lifestyle confounders were not performed (10, 20). Three small to medium-sized studies detected no independent association between maternal glycaemia and offspring fasting glucose (21, 22) or insulin resistance (21, 23). However, other studies report significant associations between maternal diabetes and offspring glucose metabolism persisting after adjustment for maternal and offspring BMI (24–26) and one when also adjusting for family history of diabetes (24). Notably, a large multicentre study including 4160 mother-child pairs, the Hyperglycaemia and Pregnancy Adverse Outcomes Follow-up Study (HAPO FUS), found associations between maternal and childhood glucose metabolism (7, 27), including levels of glycaemia below the diagnostic criteria of GDM (7). The findings were independent of family history of diabetes and maternal and offspring BMI, though not adjusted for potential lifestyle confounders. A separate follow-up study conducted from the Hong Kong HAPO cohort, also reported associations between maternal glucose levels and abnormal glucose tolerance in the offspring (11). These associations were independent of maternal pre-pregnancy BMI, children’s exercise level, and maternal and paternal diabetes status.

In the present study we aimed to investigate whether there is an association between *in utero* exposure to maternal glycaemia and offspring glucose metabolism at 9 years of age. We hypothesized that such an association would be present, consistent with the HAPO findings.

## Materials and methods

### Study design

Mother-child pairs were recruited from the RCT Training in pregnancy which was designed to investigate the effects of offering a regular exercise program during pregnancy on GDM prevalence (28). In this study no differences were observed between the intervention and control group in GDM prevalence or insulin resistance (28). Pregnant women booking appointments for their ~18-week routine ultrasound scans at St. Olavs Hospital (Trondheim University Hospital) and Stavanger University Hospital in Norway were invited to participate in the trial from 2007 to 2009. A total of 855 women were included, 660 in Trondheim and 195 in Stavanger. White women ≥ 18 years of age with a singleton live foetus who lived less than a 30-minute drive from the hospitals were eligible for inclusion. Exclusion criteria were history of diabetes or severe chronic diseases, known alcohol or drug misuse, previous severe pregnancy complications (preterm birth before 34 weeks, severe foetal growth retardation or severe preeclampsia with delivery before 34 weeks, eclampsia or HELLP syndrome), hypertension (systolic blood pressure ≥ 140 mmHg and/or diastolic blood pressure ≥ 90 mmHg) at antenatal care visit before study entry or foetal malformations, placenta previa or identified risk of preterm birth in current pregnancy (short cervix, amniotic fluid leakage or profuse vaginal bleeding with proven retroplacental hematoma) at ~18-week ultrasound scan.

The participating women were randomised to receive a 12-week exercise program or standard antenatal care. At the end of intervention (week 32–36 of pregnancy), participants were examined and underwent a 75-gram OGTT, where fasting and 2-hour glucose were analysed. Demographic and lifestyle characteristics were collected via questionnaires at two time points in pregnancy and 3 months post-partum. Pregnancy outcomes and newborn data were retrieved from medical charts after delivery.

At follow-up, questionnaire data were collected between October 2014 and December 2016 when the child was ~7 years old, by using the safe electronic solution CheckWare. Children and parents residing in Trondheim who had consented at the follow-up questionnaire to be invited to an in-person study visit, were invited to this study visit between November 2016 and December 2018, when the child was ~9 years old. The aim was assess the long-term effects on children’s health. For the women participating in this follow-up, we repeated the primary analyses from the original RCT of potential differences in GDM prevalence between the intervention and control group, using the revised WHO 2013 criteria (29), and supplemented by analysing for group differences in fasting and 2-hour glucose. If no new group differences were detected, the intervention and control group would be analysed as one cohort in this follow-up study.

Parents received written detailed information about the study. The children received a separate easy to read information letter, and parents and children were encouraged to discuss participation. The parents gave written consent on behalf of their children. The study was approved by The Regional Committee for Medical and Health Research Ethics in Central Norway (REK no. 2015/2028, application date 09-03-2016).

### Outcomes

The primary outcome in this study was offspring glucose metabolism at ~9 years of age, as expressed by fasting serum glucose and estimated insulin resistance. At the study visit, fasting blood samples were collected from the children between 07:30 and 09:00. The blood samples were collected by standard venepuncture in vacuum tubes and sat for 30 minutes at room temperature before centrifugation (3000g/4°C/10 minutes). Sera were aliquoted and stored at -80°C until further analyses. Sera from all children were analysed simultaneously for glucose and insulin with accredited analyses at St. Olavs Hospital (Trondheim University Hospital) in September 2021. Insulin resistance was estimated by HOMA-IR according to the formula (fasting glucose in mmol/L)*(fasting insulin in pmol/L)/135 (30).

### Predictors and covariates

Primary predictors were maternal fasting and 2-hour serum glucose following the OGTT at ~34 weeks of gestation (28). Covariates were chosen based on known associations with the predictors and outcomes from previous literature, and based on covariates chosen in similar studies to enable comparison with these studies. Children’s height and weight were measured at the ~9-year study visit. BMI was calculated as weight in kilograms divided by the square value of height in meters (kg/m^2^). Offspring BMI was further classified into iso-BMI categories, i.e. categories adjusted for age and sex, with the use of a calculator published by the Norwegian Institute of Public Health (31). Information about the child’s physical activity was collected by questionnaire at the ~7-year follow-up. Parents were asked if their child on average participated in moderate to vigorous physical activity ≥ 60 minutes per day (32), according to the WHO (33) and Norwegian Directorate of Health (34) recommendations for physical activity for children. Gestational age was estimated at the ~18-week routine ultrasound scan, and estimated gestational age at the OGTT was included as a covariate. Maternal pre-pregnancy BMI was calculated based on height and pre-pregnancy weight self-reported at study inclusion. Socioeconomic status was calculated based on the mother’s education and occupation reported in the questionnaire at study inclusion, according to Hollingshead Two-Factor Index of Social Position (35). Information about diabetes among the mother’s first-degree relatives (mother, father, siblings, or previous children) was collected at the questionnaire 3 months post-partum. The mother’s group allocation in the original RCT was also included as a covariate.

### Statistical analyses

Descriptive statistics for continuous variables are presented with mean, standard deviation (SD) and minimum and maximum values. Categorical variables are presented with frequencies and percentages. Histograms and Q-Q plots for continuous variables were reviewed to assess normality, and maternal insulin and HOMA-IR were logarithmically transformed before further analyses due to non-normal distribution. Independent samples t-tests were used to analyse group differences for continuous variables. For categorical variables differences were analysed using Chi-square tests or Fishers exact tests as appropriate.

Simple and multivariable linear regression analyses were performed to assess associations between outcome and exposure variables. Mother-child pairs with missing values for one or more of the predictors, covariates, or outcomes were excluded from the regression analyses. Linearity was assessed by scatterplots, and multicollinearity was evaluated by reviewing pairwise correlations and variance inflation factors. The normality of residuals was assessed with residual plots. For the multivariable regression, covariate adjustments were performed in three separate models. Model 1 adjusted for offspring age (continuous), offspring sex (girl/boy), offspring physical activity (on average ≥ 60 minutes moderate to vigorous physical activity per day: yes/no), maternal group allocation in the original RCT (intervention group/control group), maternal socioeconomic position (continuous, class 1-5), maternal age (continuous), diabetes among first degree relatives of the mother (yes/no or uncertain) and gestational age at OGTT (continuous). Model 2 adjusted for the same variables as model 1, in addition to offspring BMI (continuous). Model 3 adjusted for the same variables as model 2, in addition to maternal pre-pregnancy BMI (continuous). Statistical analyses were performed using IBM SPSS Statistics version 28. P-values less than 0.05 were considered statistically significant.

## Results

**Figure 1:**
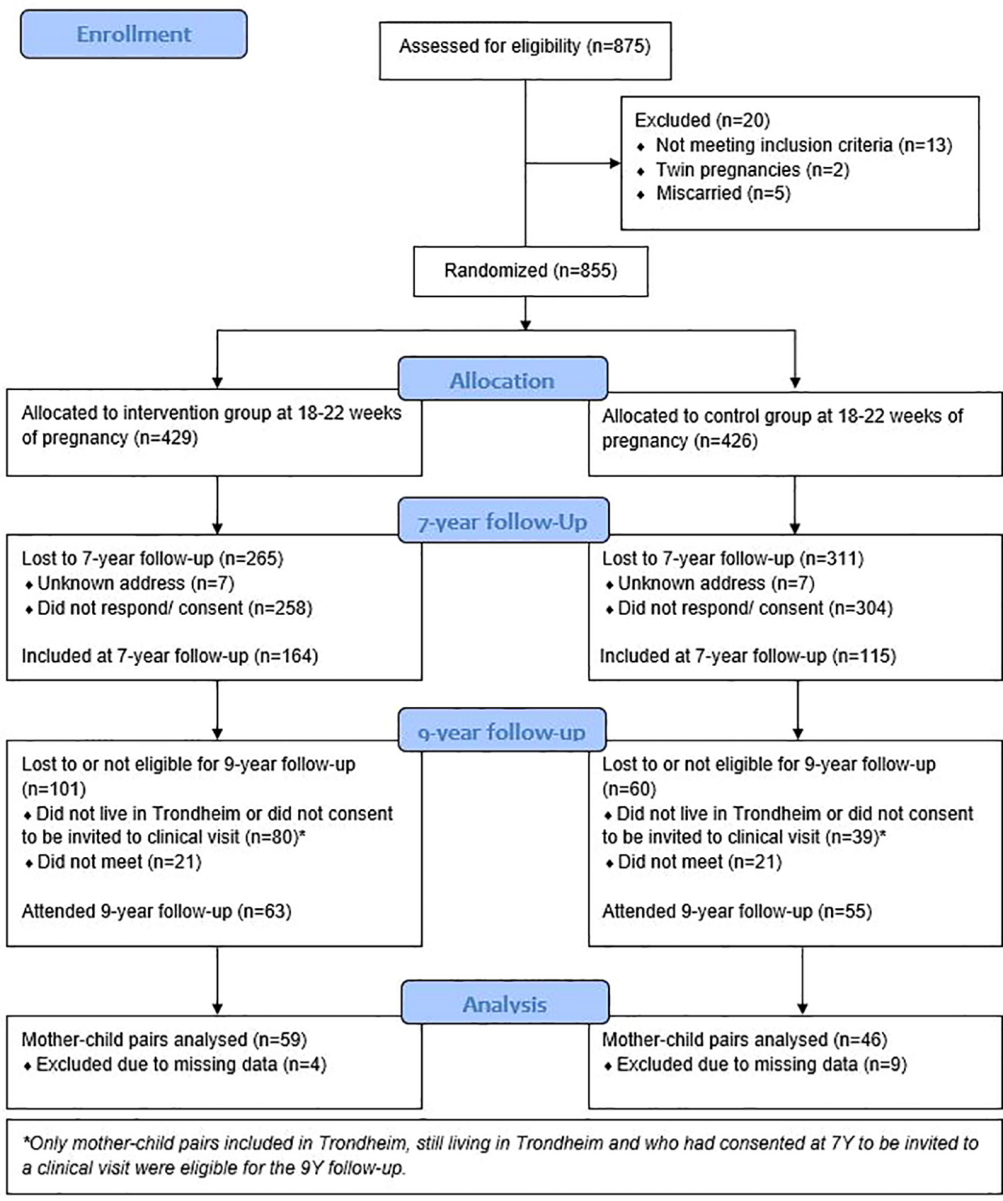
Study flow diagram.

This 9-year follow-up study included a total of 118 mother-child pairs (**Figure 1**). This accounts for 18% of the 660 mothers who were included in the original RCT in Trondheim during pregnancy, and 74% of the 160 eligible mother-child pairs who were included in the 7-year follow-up and had given their consent to be invited to participate in an in-person study visit. 13 mother-child pairs were excluded from the regression analyses due to missing values of one or more of the predictors, covariates, or outcomes. The remaining 105 (16%) mother-child pairs were included. Of these, 59 mothers (56%) were randomised to the intervention group in the original RCT. When the glucose metabolisms of the women who participated in this follow-up study were analysed, we detected no differences between the intervention and control groups in terms of the prevalence of GDM (3 of 59 (5.1%) in the intervention group and 2 of 46 (4.3%) in the control group (p=0.86)), mean fasting glucose (4.3 mmol/L in both the intervention (SD=0.3) and the control (SD=0.4) group, p=0.50), or mean 2-hour glucose (5.5 mmol/L (SD=1.4) in the intervention group and 5.7 mmol/L (SD=1.2) in the control group, p=0.60). Therefore, the intervention and control groups from the original RCT were analysed as one cohort in this follow-up study.

Mother-child pairs who did and did not participate in the 9-year follow-up are compared in **Table 1**. Characteristics were similar in the two groups, except for a slightly higher maternal age (p=0.016), higher socioeconomic status (p=0.0021), and lower fasting glucose (p=0.022) among participants. Mean maternal fasting and 2-hour glucose was 4.3 mmol/L (SD ± 0.4) and 5.6 mmol/L (SD ± 1.3), respectively. **Table 2** shows characteristics of the children at the ~9-year study visit and reported physical activity at the ~7-year follow-up. Mean fasting glucose was 4.7 mmol/L (SD ± 0.3), and mean HOMA-IR was 1.08 (SD ± 0.30).

**Table 1:** Characteristics of participants and non-participants.

Characteristic	Participants (n=118)	Non-participants (n=542)	P-value
Maternal characteristics at study inclusion ~18 weeks of gestation			
Randomised to exercise group, n (%)	63 (53%)	267 (49%)	0.42
Age, years	31.2 ± 3.6 [24-41]	30.3 ± 4.4 [19-46]	**0.016**
Education, n (%)			0.46
≤ 13 years in total	10 (8.5%)	57 (11%)	
≤ 4 years higher education	41 (35%)	211 (39%)	
> 4 years higher education	67 (57%)	274 (51%)	
Married or living with partner, n (%) ^a^	117 (99.2%)	528 (97.6%)	0.48
Socioeconomic position, class	4.1 ± [0.8]	3.8 ± [1.0]	**0.0021**
Diabetes among first degree relatives, n (%) ^b^	9 (7.6%)	43 (7.9%)	0.64
Parity, n (%)			0.48
No children	61 (52%)	313 (58%)	
1 child	40 (34%)	159 (29%)	
2 or more children	17 (14%)	70 (13%)	
Pre-pregnancy BMI, kg/m^2^ ^c^	23.1 ± 3.0 [17.5-35.9]	23.2 ± 3.3 [17.3-38.4]	0.55
Pre-pregnancy overweight or obesity (BMI ≥ 25), n (%) ^c^	25 (21%)	117 (22%)	0.89
Smoking, n (%)	2 (1.7%)	3 (0.6%)	0.22
Hypertension, n (%)	1 (0.8%)	8 (1.5%)	1.00
Maternal characteristics at third trimester exam			
Gestational age at exam, weeks ^d^	33.7 ± 2.0 [26.9-38.3]	33.7 ± 2.0 [27.0-42.6]	0.70
Fasting serum glucose, mmol/L ^e^	4.3 ± 0.4 [3.5-5.2]	4.4 ± 0.4 [3.4-6.4]	**0.022**
2-hour serum glucose, mmol/L ^f^	5.6 ± 1.3 [2.3-9.3]	5.8 ± 1.2 [3.1-9.9]	0.18
Fasting serum insulin, pmol/L ^g^	74.4 ± 1.5 [18.5-145.7]	79.6 ± 1.6 [22.1-406.7]	0.13
HOMA-IR ^h^	2.34 ± 1.52 [0.59-5.58]	2.53 ± 1.60 [0.59-15.07]	0.11
GDM, n (%) ^f^	5 (4.4%)	26 (5.8%)	0.55
Neonatal characteristics			
Gestational age at birth, weeks ^a^	40.1 ± 1.3 [34.7-42.3]	40.0 ± 1.7 [27.0-42.6]	0.39
Birth weight, g ^a^	3565 ± 464 [1940-4830]	3509 ± 546 [850-4930]	0.30
Female sex, n (%) ^a^	61 (52%)	260 (48%)	0.47
Vaginal delivery, n (%) ^i^	104 (88%)	482 (89%)	0.68

BMI, body mass index; HOMA-IR, Homeostatic model assessment for insulin resistance; GDM, Gestational diabetes mellitus defined by the 2013 WHO criteria as fasting glucose ≥ 5;1 mmol/L or 2-hour glucose ≥ 8;5 mmol/L; Hypertension defined as systolic blood pressure ≥ 140 mmHg and/or diastolic blood pressure ≥ 90 mmHg.

Data are mean ± SD [min - max] or n (%)

a : Data were missing for 1 woman in the non-participant group.

b : Data were missing for 3 women in the participant group, and for 75 women in the non-participant group.

c : Data were missing for 6 women in the non-participant group.

d : Data were missing for 4 women in the participant group, and for 75 women in the non-participant group.

e : Data were missing for 4 women in the participant group, and for 89 women in the non-participant group.

f : Data were missing for 4 women in the participant group, and for 96 women in the non-participant group.

g : Data were missing for 6 women in the participant group, and for 76 women in the non-participant group.

h : Data were missing for 6 women in the participant group, and for 90 women in the non-participant group.

i : Data were missing for 3 women in the non-participant group. The bold values are statistically significant; however, they may preferably be reformatted back to normal text as the information is not necessary to include in this table.

**Table 2:** Characteristics of the children at follow-up.

Characteristic	Children (n=118)
Questionnaire at ~7 years	
Daily MVPA: 1 h or more, n (%) ^a^	75 (66%)
Study visit at ~9 years	
Age, years	9.0 ± 0.4 [8.1-10.0]
Height, cm	137 ± 6 [121-153]
Weight, kg	31 ± 4 [22-46]
BMI, kg/m^2^	16.6 ± 1.7 [12.8-24.3]
Iso-BMI categories, n (%)
Underweight (isoBMI ≤ 18.4)	5 (4.2%)
Normal weight (isoBMI 18.5-24.9)	106 (89.8%)
Overweight (isoBMI ≥ 25)	7 (5.9%)
Serum glucose, mmol/L ^b^	4.7 ± 0.3 [3.5-6.4]
Serum insulin, pmol/L	31.5 ± 8.4 [10.0-56.4]
HOMA-IR ^b^	1.08 ± 0.30 [0.31-1.80]

BMI, body mass index, Iso-BMI, BMI adjusted for age and sex, HOMA-IR, Homeostatic model assessment for insulin resistance; MVPA, moderate to vigorous physical activity.

Data are mean ± SD [min - max] or n (%)

a : Data were missing for 5 children.

b : Data were missing for 4 children.

Model diagnostics for linear regression analyses showed a good fit. Scatterplots indicated linearity between predictors and outcomes, and residual plots indicated reasonably normal distributions. Collinearity assessment showed pairwise correlations ranging between 0.0039 and 0.28 for covariates, except for the correlation between maternal fasting glucose and maternal pre-pregnancy BMI, which was 0.47. Although higher than ideal for the regression analyses, it was acceptable, and variance inflation factors were satisfactory; 1.31 for maternal fasting glucose and 1.36 for maternal pre-pregnancy BMI.

Results of the linear regression analyses are presented in **Tables 3**, **4**. The analyses examined associations of maternal fasting and 2-hour glucose with offspring fasting glucose (**Table 3**) and offspring insulin resistance (**Table 4**). In the unadjusted analysis, a positive, though borderline significant association was observed between maternal and offspring fasting glucose. Adjusting for possible confounders and other covariates in model 1, and further adjustment for offspring BMI in model 2, strengthened the association. Adjusting for pre-pregnancy BMI in model 3, weakened the association. Other investigated associations were not significant in any of the models.

**Table 3:** Associations between maternal glucose and offspring fasting glucose (n=105 mother-child pairs).

	Crude		Model 1		Model 2		Model 3	
	Beta (95% CI)	P-value	Beta (95% CI)	P-value	Beta (95% CI)	P-value	Beta (95% CI)	P-value
Maternal fasting glucose, mmol/L	0.17 (-0.011 to 0.35)	0.066	0.18 (-0.0013 to 0.36)	0.052	0.18 (-0.00027 to 0.37)	0.050	0.15 (-0.053 to 0.36)	0.14
Maternal 2-hour glucose, mmol/L	0.024 (-0.027 to 0.075)	0.35	0.016 (-0.037 to 0.069)	0.56	0.016 (-0.038 to 0.070)	0.56	0.010 (-0.044 to 0.064)	0.71

CI, confidence interval.

Model 1: adjusted for maternal group allocation in the original RCT, maternal age, maternal socioeconomic position, gestational age at OGTT, diabetes among first degree relatives of the mother (yes/no or uncertain), offspring age, offspring sex and offspring physical activity (on average ≥ 60 minutes moderate to vigorous physical activity per day: yes/no).

Model 2: model 1, additionally adjusted for offspring BMI.

Model 3: model 2, additionally adjusted for maternal pre-pregnancy BMI.

**Table 4:** Associations between maternal glucose and offspring insulin resistance (HOMA-IR) (n=105 mother-child pairs).

	Crude		Model 1		Model 2		Model 3	
	Beta (95% CI)	P-value	Beta (95% CI)	P-value	Beta (95% CI)	P-value	Beta (95% CI)	P-value
Maternal fasting glucose, mmol/L	0.063 (-0.097 to 0.22)	0.44	0.078 (-0.087 to 0.24)	0.35	0.080 (-0.087 to 0.25)	0.34	0.042 (-0.14 to 0.23)	0.66
Maternal 2-hour glucose, mmol/L	0.014 (-0.031 to 0.059)	0.53	0.016 (-0.031 to 0.064)	0.50	0.017 (-0.032 to 0.065)	0.49	0.013 (-0.036 to 0.061)	0.61

CI, confidence interval.

Model 1: adjusted for maternal group allocation in the original RCT, maternal age, maternal socioeconomic position, gestational age at OGTT, diabetes among first degree relatives of the mother (yes/no or uncertain), offspring age, offspring sex and offspring physical activity (on average ≥ 60 minutes moderate to vigorous physical activity per day: yes/no).

Model 2: model 1, additionally adjusted for offspring BMI.

Model 3: model 2, additionally adjusted for maternal pre-pregnancy BMI.

## Discussion

### Main findings

In this study, assessing 105 mother-child pairs, we observed a borderline significant positive association (beta=0.18, 95% CI [-0.00027, 0.37], p=0.050) between maternal and offspring fasting glucose when adjusted for potential confounders (model 2). The interpretation of this is that a 1 mmol/L increase in maternal fasting glucose, keeping everything else constant, would lead to a 0.18 mmol/L increase in offspring fasting glucose. We did not find significant associations between maternal fasting glucose and offspring insulin resistance, or between maternal 2-hour glucose and offspring fasting glucose or insulin resistance.

### Strengths and limitations

This study has two main strengths in its investigation of the association between maternal and offspring metabolism. First, we examined serum levels of glucose and insulin which are objective measures of the offspring outcomes and maternal exposures. Second, a variety of relevant covariates were collected through study visits and questionnaires during pregnancy and at 7- and 9-year follow-ups, including both potential hereditary and lifestyle confounding factors.

There are inherent limitations to the analysis. The sample size is small, which limits the study’s power to demonstrate associations. Though associations between investigated exposures and outcomes were positive, confidence intervals were wide, and p-values did not meet the set significance level at < 0.05. Moreover, the study was not originally designed to examine the long-term effects of maternal hyperglycaemia in pregnancy, and no a-priori power calculation was performed. Though the study is generally well adjusted for confounders, we did not have access to relevant paternal factors, such as diabetes, BMI, and socioeconomic status.

Offspring fasting blood samples were analysed in this study. It is possible that glucose tolerance (1- or 2-hour glucose), obtained following a 75-gram glucose load, would be more sensitive in identifying abnormal glucose metabolism in children, as argued in a systematic review by *Kawasaki et al.* (10). However, the HAPO FUS detected associations between maternal 2-hour glucose and both offspring fasting and 2-hour glucose (7). The disadvantage of performing glucose tolerance tests is the increased discomfort for the participating children, and in this study, we therefore preferred fasting blood samples.

The OGTT was performed at ~34 weeks of gestation in the present study, in contrast to many other studies, and screening programs in most countries, where testing in week 24-28 is recommended. This is of importance when comparing results with other studies, as glucose metabolism changes during a normal pregnancy, with decreasing fasting glucose and increasing non-fasting glucose and insulin resistance with advancing gestation (36).

It is possible that the present study might be exposed to some degree of selection bias, in favour of healthier mother-child pairs. 74% of the eligible mother-child pairs from the 7-year follow-up participated in this 9-year follow-up study. However, only 18% of the mother-child pairs included in the original study in Trondheim participated in the present follow-up study. Participating mothers had higher socioeconomic position and lower third trimester fasting glucose than non-participants, though other characteristics were similar between the groups. It is also reasonable to assume that women who agreed to participate in the initial RCT might be healthier than the general population, as it was an exercise study. Compared with data from the medical birth registry of Norway (MBRN) in 2021 (37) the women in our study had slightly better metabolic health. The percentage of women with GDM (4.4% in this study and 6.3% in MBRN) and pre-pregnancy overweight or obesity (BMI ≥ 25) (21% in this study and 38% in MBRN) was lower in our study, even though MBRN utilized slightly stricter GDM criteria and OGTTs were performed earlier in pregnancy. Compared to the HAPO FUS participants (7), the women in the current study had slightly lower mean fasting glucose (4.3 mmol/L in this study and 4.5 mmol/L in HAPO FUS), lower mean 2-hour glucose (5.6 mmol/L in this study and 6.1 mmol/L in HAPO FUS), and a considerably lower percentage had GDM, 4.4%, vs 14.1% in HAPO FUS, using the same GDM criteria (29). Notably, the glucose analyses were performed at a mean gestational age of 28 weeks in the HAPO study, i.e. 6 weeks earlier than in the present study, indicating that the genuine difference in metabolic health might be even larger than displayed by these numbers. The present study and the HAPO FUS had different ethnic compositions, which may also have influenced the results.

Comparison with the ungKan3 study (38), reporting health and physical activity of Norwegian children in 2018, indicates that the children as well as the mothers in the present study are healthier than the Norwegian average. A considerably lower percentage in the present study were overweight or obese (6% in this study and 21% in ungKan3), both studies with a mean participant age of 9 years. The children in our study were, however, equally or slightly less physically active, with 66% in total meeting the WHO recommendation of ≥ 60 minutes of moderate to vigorous physical activity per day, vs 64% of the girls and 81% of the boys in the u*ngKan3* study. This difference might be caused by different measuring techniques (accelerometers in UngKan3 vs reported by parents in our study), as it has been shown that parents tend to under-report children’s physical activity (39). To conclude, the study population of both mothers and children seemed to be healthier than average and relatively homogeneous, which might have decreased the chance of detecting potential associations in our analyses. It may also decrease the generalizability of our findings to the general population. However, it would be unexpected for detected associations to be stronger when investigating a healthier study population.

### Interpretation

In the HAPO FUS, significant associations between maternal and offspring glucose levels were detected, which were suggested by the authors to be clinically important, possibly contributing to the increasing prevalence of type 2 diabetes in children (7). In the present study comparable beta-estimates (in fully adjusted models, with child BMI, but without maternal BMI) were similar or higher than in the HAPO FUS. For the association between maternal and offspring fasting glucose, our beta-estimate of 0.18 (95% CI [-0.00027, 0.37]) was higher than in the HAPO FUS (0.042, 95% CI [0.031, 0.054]), and for the association between maternal 2-hour glucose and offspring fasting glucose, beta-estimates were similar (0.016 in the present study and 0.014 in the HAPO FUS). However, confidence intervals were wider in our study, as expected with a smaller sample size. Although borderline significant, our observed association between maternal and fasting glucose supports and aligns with the HAPO FUS findings.

The HAPO FUS observed the strongest associations between maternal and offspring fasting glucose, and maternal and offspring 2-hour glucose. The authors argued that this suggested that hereditary factors, not captured by family history, contributed to the associations (7). Consistent with HAPO FUS, the strongest association in our study was the association between maternal and offspring fasting glucose.

The children in our study were examined at ~9 years of age. There are indications that associations between maternal and offspring glucose metabolism might be more evident in older children. Most studies detecting significant associations have examined children older than in the current study (7, 16, 24–26, 40). The systematic review by Kawasaki et al. discovered significant associations between GDM and offspring fasting glucose among 20-year-olds, but not among 15-year-olds or 7-10-year-olds (10). However, the Hong Kong HAPO cohort found significant associations between maternal glucose levels and offspring risk of abnormal glucose tolerance among 7-year-old children (11).

When evaluating the relationship between maternal and offspring glucose metabolism it is relevant to assess whether an association is mediated by the *in utero* environment or by the influence of hereditary factors or postnatal environment. To assess the *in utero* exposure effects, the analyses in models 1-3 in this study were adjusted for potential confounding factors related to heredity or similar lifestyle. Diabetes among first-degree relatives of the mother was included in the analyses for this purpose. Socioeconomic status may be associated with lifestyle factors of both mother and child, which in turn may affect their respective glucose metabolisms, and several studies support the relationship between socioeconomic status and child metabolic health (41, 42). Physical activity of the children is also known to affect their glucose metabolism (43, 44), and may be associated with maternal glucose through similar mechanisms as socioeconomic status.

Previous literature does not provide clear evidence on how maternal BMI affects the relationship between maternal and offspring glucose metabolism. BMI is an established risk factor for hyperglycaemia in pregnancy (45). Several studies have also detected associations between pre-pregnancy BMI and offspring glucose levels, however, not after adjustment for offspring BMI (22, 26, 46, 47). This indicates that the relationship between pre-pregnancy BMI and offspring glucose levels is largely mediated by offspring BMI, and it is therefore not necessary to adjust for both maternal and offspring BMI. In the current study, analyses without pre-pregnancy BMI were performed in models 1 and 2, and the model 2 analyses were adjusted for offspring BMI to minimise the risk of confounding. Offspring BMI was included in the model 2 analyses with the additional purpose of examining if offspring BMI had a role as a mediator in the relationship between maternal and offspring glucose metabolism. In the HAPO FUS (7) and in the *Effect of Preeclampsia On Cardiovascular Health* (EPOCH) study (26), results were not attenuated when adjusting for offspring BMI, indicating that the associations were mediated by other mechanisms. Though insufficient to firmly conclude, the associations in our study were not attenuated when adjusting for offspring BMI, thus pointing in the same direction. Most studies investigating similar exposures and outcomes as this study have included maternal BMI or overweight in their analyses (7, 11, 21–24, 27), and maternal pre-pregnancy BMI was included in the model 3 analyses in our study to enable comparison with relevant studies. The model 3 analyses show that the associations were attenuated when adjusting for maternal BMI, but the reason cannot be determined with certainty. It is possible that the previously mentioned collinearity between maternal BMI and maternal fasting glucose, though below commonly used cut-of values, is affecting the precision of the model 3 analyses. The attenuated results might also be caused by maternal BMI mediating the association between maternal and offspring glucose levels.

In the current study, adjustments were also performed for selected additional covariates. Gestational age at the time of the OGTT was included as a covariate because of the known physiological changes in glucose metabolism over the course of pregnancy (36). Maternal age was considered a possible confounder because it affects the risk of hyperglycaemia in pregnancy (45) and might affect the long-term health of the offspring (48). Smoking during pregnancy and maternal hypertension were also assessed to be interesting covariates, but not included in the final analyses due to the few cases.

Other studies investigating associations between maternal and offspring glucose metabolism are generally less comprehensively adjusted than the present study, missing adjustments for hereditary and/or lifestyle confounding factors (7, 16, 21, 24–27, 40, 49). However, most of the studies conclude that *in utero* exposure probably constitutes part of the association between maternal and offspring glucose metabolism. This hypothesis is also supported by studies of Pima Indians, comparing the risk of diabetes between siblings born before and after their mother was recognised as having diabetes (50). Furthermore, it is important to note that the Hong Kong HAPO cohort found that maternal glucose levels were associated with the risk of offspring abnormal glucose tolerance, when adjusted for both lifestyle and hereditary factors (11). Though borderline significant and with a lower sample size, the results in the present study were not attenuated by adjustment for confounders, thus supporting previous studies.

### Future perspectives

Previous literature and our study indicate that maternal hyperglycaemia negatively impact the offspring’s long-term health. Optimising metabolic health before conception and during pregnancy may therefore lead to offspring with improved metabolic health, and this should be a focus point for improving public health. Further studies should be performed to confirm the associations, with greater power and adjustment for as many covariates. Moreover, the effects of maternal interventions on long-term offspring outcomes are not yet well documented and future high-quality studies are needed to address this gap.

### Conclusion

In the present study, assessing a small group of healthy mothers and children at low risk of metabolic diseases, we observed a borderline significant positive association between maternal and offspring fasting glucose, which persisted after adjustment for potential hereditary and lifestyle confounding factors. Our findings support other similar studies and highlight that improving the metabolic health of pregnant women, and women of childbearing age, should remain a key public health priority.

## Data availability statement

The raw data supporting the conclusions of this article will be made available by the authors, without undue reservation.

## Ethics statement

The studies involving humans were approved by The Regional Committee for Medical and Health Research Ethics in Central Norway. The studies were conducted in accordance with the local legislation and institutional requirements. Written informed consent for participation in this study was provided by the participants’ legal guardians/next of kin.

## Author contributions

SN: Formal Analysis, Writing – original draft, Writing – review & editing. AS: Conceptualization, Data curation, Methodology, Supervision, Writing – review & editing. KE: Conceptualization, Data curation, Methodology, Writing – review & editing. TB: Data curation, Methodology, Writing – review & editing. US: Conceptualization, Data curation, Methodology, Writing – review & editing. KS: Methodology, Project administration, Writing – review & editing. SM: Conceptualization, Funding acquisition, Investigation, Methodology, Project administration, Writing – review & editing. SS: Conceptualization, Data curation, Formal Analysis, Funding acquisition, Investigation, Methodology, Project administration, Supervision, Writing – original draft, Writing – review & editing.
